# Editorial: Chronic inflammation and pharmacological interventions in cardiovascular diseases

**DOI:** 10.3389/fphar.2022.993569

**Published:** 2022-08-24

**Authors:** Xiaoping Wang, Min Zhang, Xianwei Wang

**Affiliations:** ^1^ Department of Cardiology, The First Affiliated Hospital Xinxiang Medical University, Weihui, China; ^2^ Department of Human Anatomy and Histoembryology, Xinxiang Medical University, Xinxiang, China; ^3^ School of Cardiovascular and Metabolic Medicine & Sciences, King’s College London BHF Centre of Research Excellence, London, United Kingdom

**Keywords:** chronic inflammation, cardiovascular diseases, pharmacological interventions, cell-based therapies, cellular and molecular mechanisms

## Introduction

Cardiovascular diseases (CVDs) are a group of complex and multifactorial disorders and their pathogenesis is still not completely understood. It is recognized that inflammation, especially the chronic inflammation is a common pathogenesis of many CVDs, such as atherosclerosis, myocardial infarction and stroke. Continuous inflammation causes a series of pathological changes of hearts and blood vessels. Clinical trials and basic studies have shown that inflammatory inhibition by pharmacological and other interventions can markedly reduce the degree of pathological changes of hearts or blood vessels, and decrease the morbidity and mortality of CVD events. Thus, the advance of therapies direct or indirect modulating chronic inflammation is an important approach for the prevention and treatment of CVDs.

Pharmacological interventions are the primary therapeutic approaches for CVDs. In the past few decades, plenty of drugs for different targets including anti-inflammatory agents have been developed and used to prevent and treat different CVDs in the clinic. In the past few years, the flood of new drugs including chemical-based drugs and natural herbal products have provided more choices to the treatment of CVDs. Recently, biotherapies and molecular targeted therapies utilizing cell-based products such as stem cells, exosomes, immune cells, cytokines, peptides and monoclonal antibodies have also been used to treat CVDs, and some of them specifically target to inflammation. The purpose of this Research Topic was to provide interested readers with new advances on prevention and treatment of CVDs, especially on the new therapies targeting chronic inflammation.

**FIGURE 1:**
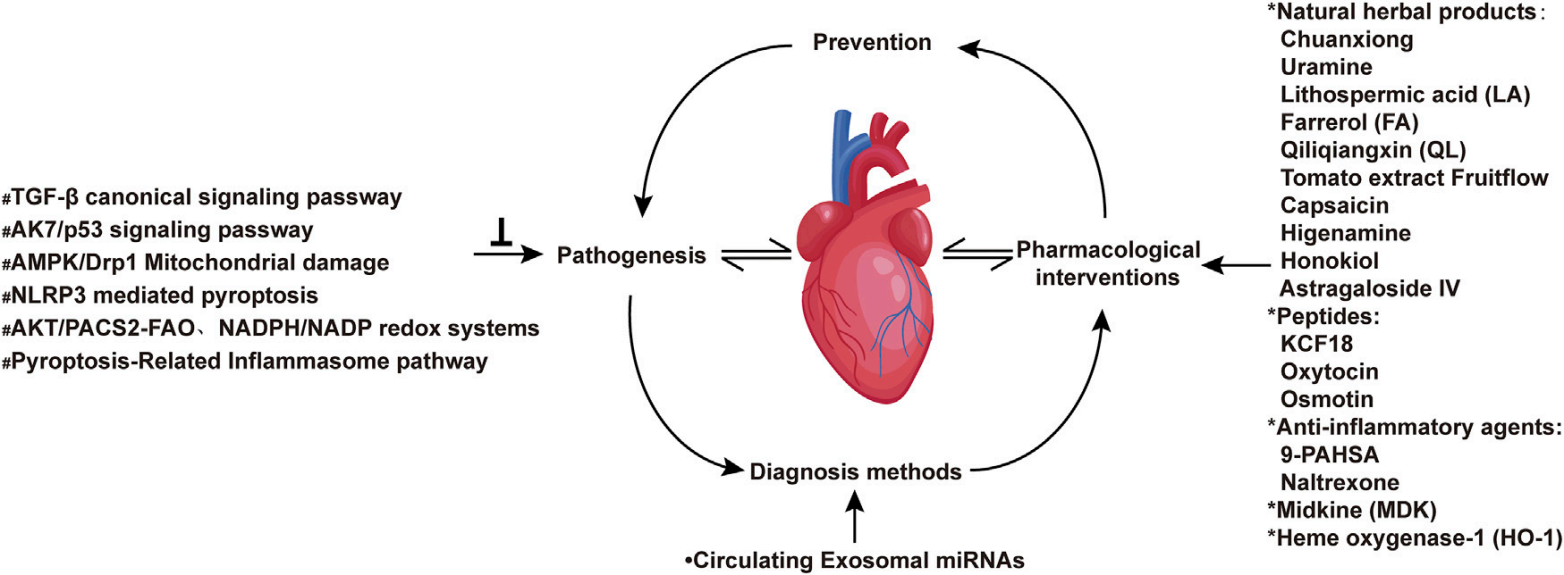
Inflammatory signaling pathways and pharmacological interventions in cardiovascular diseases. *Different types of drugs that interfere with cardiovascular inflammation covered in the research theme; **•**New diagnostic method for cardiovascular diseases; ^**#**^Cellular and molecular mechanisms involved in chronic inflammation in cardiovascular diseases.

The Research Topic themed “Chronic inflammation and pharmacological interventions in cardiovascular diseases” presents a series of articles that highlight the latest studies and strategies that overcome current obstacles in treating chronic inflammation and pharmacological interventions in cardiovascular diseases. This issue collates 30 selected peer-reviewed articles (including 20 original research articles and 10 reviews) covering molecular mechanisms of therapeutic targets, drug intervention targets, and novel diagnostic approaches to cardiovascular diseases (Figure 1).

## Related inflammatory pathways in Cardiovascular Diseases

Atherosclerosis is a serious clinical manifestation of cardiovascular disease (Soehnlein and Libby, 2021), which is the leading cause of premature death in humans (Valanti et al., 2021). Chronic inflammatory response is an important risk factor for the initiation and development of atherosclerosis (Yang et al., 2019), but the inflammatory molecular mechanisms in the occurrence and development of atherosclerotic plaques are not well understood. There are several articles in this Research Topic showed that some chronic inflammatory pathways play critical roles in cardiovascular diseases. Although increasing evidence indicates that genetic factors, particularly the transforming growth factor *β* (TGF-β) signaling pathway is involved in the development of aortic aneurysms (AAs), the specific action of TGF-β signaling in AAs remains controversial (Thatcher, 2016; Tzavlaki and Moustakas, 2020). The review by Chen and Chang focused on the role of canonical TGF-β signaling pathway related to core genes such as TGFBR1,TGFBR2, SMAD2, SMAD3, SMAD4, and SMAD6 in aortic diseases. This review further clarified that the activation of classical TGF-β signaling pathway is a determinant of a series of aortic diseases. Wang et al. revealed that liver kinase B1 (LBP1), a serine threonine kinase, played an important role in arteriosclerosis by regulating vascular macrophages after phosphorylation of activating adenosine monophosphate-activated protein kinase (AMPK). Periodontitis is a common chronic disorder that involves oral microbe-related inflammatory bone loss and local destruction of the periodontal ligament and is a risk factor for atherosclerosis (Naderi and Merchant, 2020). Periodontal pathogens produce pathogen associated molecular patterns (PAMPs), including lipopolysaccharide (LPS), PGN, and CpG DNA. Periodontal infection activates neutrophils to form neutrophil extracellular traps (NETs), which together with high mobility group box-1 (HMGB1) and alarmins released by damaged periodontal cells constitute damage-associated molecular patterns (DAMPs) (Gu and Han, 2020). Zhu et al. reported that PAMPs and DAMPs could activate excessive innate immunity by acting on Toll-like receptors (TLRs) and NOD-like receptors (NLRs) in arterial tissue, leading to foam cell formation, endothelial cell and vascular smooth muscle cell dysfunction, and promoting the massive release of inflammatory factors, which all contribute to atherosclerosis.

Diabetic cardiomyopathy (DCM) is one of the serious complications of diabetes, and increasing evidence supports that myocardial inflammation is a key player in the development of DCM (Dillmann, 2019). Pyroptosis is a type of programmed cell death that involves the release of cell contents and inflammatory mediators upon activation, leading to a powerful inflammatory response (Wang et al., 2020). While accumulating evidence implicates pyroptosis as a critical contributor to myocardial inflammation in the progress of DCM (Yang et al., 2019; Zeng et al., 2020), the molecular mechanisms of cell pyroptosis and its involvement in DCM are not fully understood. Cai et al. reviewed the recent progress in this research field and discussed three main signaling pathways to potentially trigger DCM: 1) Toll like receptor 4 (TLR4)/nuclear factor kappa B (NF-kB) inflammasome/NOD-like receptor, pyrin domain-containing 3 (NLRP3) inflammasome signaling pathway; 2) AMPK/ROS/TXNIP/NLRP3 inflammasome signaling pathway; and 3) AMPK/SIRT1/Nrf2/HO-1/NF-kB inflammasome signaling pathway, all of which could be the possible therapeutic targets for the treatment of DCM in the future. Meanwhile, Gao et al. analyzed the beneficial effect of Cycloxanthine D (CVB-D) on cardiomyocyte pyroptosis associated with DCM, and explored its molecular regulatory mechanism. Their results demonstrated that CVB-D could ameliorate DCM by inhibiting cardiomyocyte pyroptosis via NLRP3 signaling *in vivo* and *in vitro*. These studies once again suggest that the signaling pathways of inflammatory response induced by cell pyroptosis play important roles in cardiovascular diseases.

## Natural drug intervention in cardiovascular diseases

Pharmacological interventions are the main treatment for cardiovascular diseases, and a variety of drugs have been developed for the prevention and treatment of different cardiovascular diseases. Chinese medicine has its unique advantages since it treats patients holistically as well as individually based on the personalised interventions (Fu et al., 2021). In this area, scholars reviewed the effects of traditional Chinese medicine Chuanxiong on cardiovascular and cerebrovascular diseases (Li et al.) and Uramine on heart diseases (Wen et al.). For example, the traditional Chinese medicine is effective to treat heart failure by targeting heat shock proteins (Wen et al.), as well as to treat atherosclerosis and other cardiovascular diseases by promoting blood circulation together with aspirin (Zhao et al.). Some researchers have further explored the signaling pathways of its action. Zhang et al. reported that lithospermic acid (LA) protected against myocardial ischemia-reperfusion (MI/R)-induced cardiac injury by promoting eNOS and Nrf2/HO-1 signaling via phosphorylation of AMPKα. Zhou et al. elucidated the molecular mechanism of action of a Chinese traditional medicine Farrerol (FA) in ischemia-reperfusion (I/R) injury. Their findings showed that FA indirectly protected cardiomyocytes by targeting macrophages, without relying on Nrf2-dependent or autophagy-dependent pathways, but indirectly protected cardiomyocytes through the inhibition of the interaction of NEK7 and NLRP3, thereby abolishing the assembly and activation of NLRP3 inflammasome, resulting in an effective inhibition to alleviate MI/R injury. Liu et al. investigated the effects and mechanisms of Honokiol (HL) in a rabbit atrial fibrillation (AF) model and found that the activation of Sirt3-dependent pathway participated in atrial metabolic remodeling during AF, which could be inhibited by HL via regulating the Sirtuin-3 (Sirt3) dependent pathway. Sirt3 is widely recognized to be critically involved in diverse cardiovascular diseases including cardiac and vasculature remodeling (Dikalova et al., 2020; Tomczyk et al., 2022). Lu et al. reported that qiliqiangxin (QL) ameliorated ventricular remodeling and heart failure (HF) to some extent in rats by modulating the gut microbiota and NLRP3 inflammasome. Based on network pharmacology and experimental pharmacological analysis, Jing et al. identified that Astragaloside IV, a main active compound from *Astragalus membranaceus*, could be a promising agent to improve l-NAME-induced hypertensive heart disease partly via modulation of eNOS and oxidative stress. In addition, Zhang et al. found that water-soluble tomato extract fruitflow could inhibit platelet activation, which is beneficial to people who are at risk for platelet hyperactivity-associated thrombosis.

## Molecular targeted therapy in cardiovascular diseases

Recently, molecular targeted therapies have been broadly applied to treat CVDs, which include protein and peptide drugs, nucleic acid drugs, and gene editing technologies (Xu and Song, 2021). Apart from Chinese herbal medicine as stated above, the more targeted therapies including peptides have also been studied in the treatment of cardiovascular diseases and chronic inflammation. Yang et al. demonstrated that Oxytocin (OT) ameliorated cardiac hypertrophy by inhibiting PI3K/Akt pathway via lncRNA GAS5/miR-375-3p/KLF4 axis. Based on in Silico analysis, Chang et al. highlighted the importance of the multitarget therapeutic peptide KCF18 which could alleviate inflammation by blocking the interactions of TNF-α, IL-6, and IL-1β with their cognate receptors, thus reducing the translocation of NF-κB and decreasing the inflammatory gene expressions. By lowering the release of cytokines in plasma and directly affecting vascular cells, KCF18 was shown to significantly attenuate vascular inflammation. The property of KCF18 to prevent inflammation may hold a promise as a new treatment strategy for sepsis and other inflammatory vascular diseases.

## New diagnostic methods in cardiovascular diseases

Ischemic stroke is a common serious disease caused by arteriosclerosis (Banerjee and Chimowitz, 2017). Accurate and timely diagnosis of ischemic stroke is the key for the subsequent treatment. Prior studies on biomarkers for ischemic stroke have focused on proteins in plasma such as neuron-specific enolase and interleukin (Tiedt, Prestel et al., 2017). Recently, the applications of miRNAs as the sensitive biomarkers have also attracted significant research attention in a variety of disease settings (Rupaimoole and Slack, 2017). However, there is scarcely any research to investigate the potential of exosome miRNAs as the diagnostic biomarkers for ischemic stroke. In this issue, Niu et al. reported for the first time that circulating exosome miRNAs including miR-369-3p, miR-493-3p, miR-379-5p, and miR-1296-5p could be the novel biomarkers with higher efficiency compared to conventional plasma factors in the diagnosis of large-artery atherosclerosis stroke (LAA). This is of great interest considering the method as time saving and cost saving. The authors hoped that exosomal miRNAs as new biomarkers can be applied for the prognosis analysis of LAA stroke, which may be helpful to improve the quality of life of stroke patients in the future.

## Summary

The pathogenesis of cardiovascular diseases is complex and interlinked, involving central mechanisms such as cardiomyocyte hypertrophy and death as well as systemic mechanisms such as chronic inflammation. Over last decades, significant evidence from both pre-clinical and clinical studies strongly indicates that targeting inflammation either locally or globally is an effective means for the prevention and treatment of cardiovascular diseases. Here, it is exciting to witness the latest advance, the beautiful works published in this issue elucidate some novel molecular mechanisms, identify some promising peptides, cytokines and natural products, and therefore provide the new insight into the pathogenic role of chronic inflammation in cardiovascular diseases. It is expected that this will continue to be a hot research area, the more detailed dissection of mechanisms and evaluation of the therapeutic potential by targeting inflammation undoubtedly has great translational significance for the treatment of cardiovascular diseases.
